# Maintaining immobilisation devices on trauma patients during CT: a feasibility study

**DOI:** 10.1186/s13049-017-0428-3

**Published:** 2017-08-23

**Authors:** Pål Johan Stokkeland, Erlend Andersen, Maria Myhre Bjørndal, Arne Morten Mikalsen, Sindre Aslaksen, Per Kristian Hyldmo

**Affiliations:** 1 Department of Radiology, Sørlandet Hospital Kristiansand, Kristiansand, Norway; 2 Clinic for Medical Services, Sørlandet Hospital Kristiansand, Kristiansand, Norway; 3 The Norwegian Air Ambulance Foundation, Drøbak, Norway; 4 Trauma Unit, Sørlandet Hospital, Kristiansand, Norway

**Keywords:** Trauma, Spinal immobilisation, Vacuum mattress, Plastic valve, Anthropomorphic phantom, Computed tomography, Image quality, Artefacts

## Abstract

**Background:**

To reduce the possibility of secondary deterioration of spinal injuries, it is desirable to maintain the spinal immobilisation that is applied in the prehospital setting throughout computed tomography (CT) scanning. A previous study found that metallic components within the inflation valve of the vacuum mattresses caused CT artefacts. The aim of our study was to investigate the effect of vacuum mattresses with plastic valves on CT artefacts, the radiation dose, and noise compared to a trauma transfer board and the spine boards currently used in our trauma system.

**Methods:**

We scanned an anthropomorphic whole body phantom with different immobilisation devices on a 128-slice CT scanner using the standard polytrauma CT-protocol at our institution. The phantom was scanned without any immobilisation device and with three different vacuum mattresses, two spine boards, and one trauma transfer board. Two radiologists independently assessed the artefacts. Agreement between the two radiologists was measured using the kappa coefficient. The radiation dose and noise were assessed.

**Results:**

One spine board produced major artefacts due to its metal components. One of the vacuum mattresses resulted in artefacts that impaired clinical judgement. Otherwise, the artefacts predominantly did not impede clinical judgement and were mainly subtle. One of the vacuum mattresses resulted in no artefacts that affected clinical judgement. The overall inter-rater agreement was substantial (0.86, kappa 0.77). We did not observe any artefacts due to plastic valves. The mean CT radiation dose was slightly higher for two of the devices in the head series than that for the trauma transfer board, used as the standard in our system. Only marginal differences were noted for the other devices and series. Small differences in image noise were found between the devices.

**Conclusions:**

Our results indicate that it is feasible to maintain some vacuum mattresses with plastic valves on trauma patients during CT scanning. The tested mattresses did not result in a considerably increased radiation dose or artefacts that hampered clinical judgement. One of the tested vacuum mattresses produced no artefacts that hampered clinical judgement whatsoever.

## Background

**Fig. 1:**
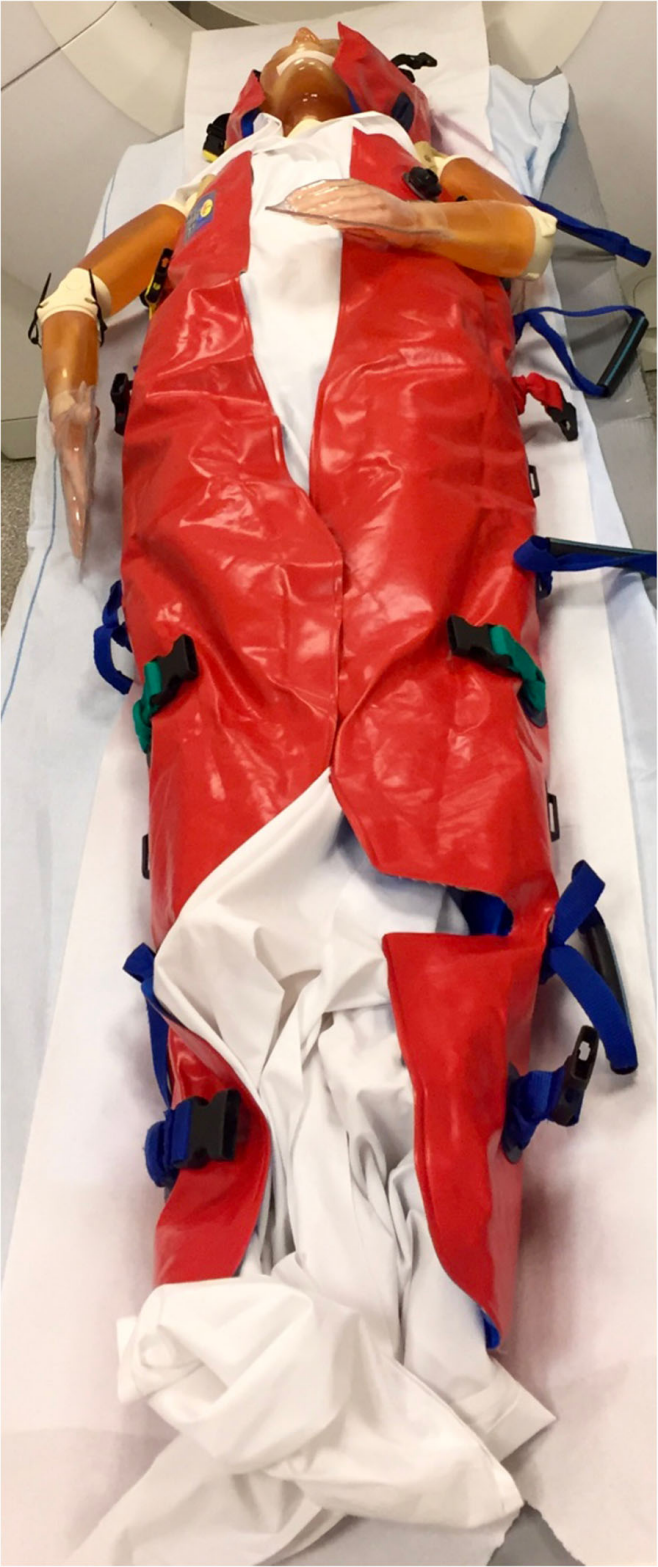
The phantom in a vacuum mattress

In trauma care, different devices are used to restrain motion in possible spinal injury cases to prevent secondary deterioration [1, 2]. Some examples include semi-rigid cervical collars and spine boards. However, a growing concern exists that these devices may harm the patient [3, 4]. A vacuum mattress is a deflatable whole body mattress containing small plastic beads. The mattress is moulded around the patient and deflated, leaving the device hardened to reduce spinal motion (Fig. 1). The vacuum mattress is gaining popularity in emergency medical services systems in Europe and is thought to offer equal or superior support and comfort compared to spine boards [5–7]. In our trauma system, spine boards and vacuum mattresses are normally removed in the emergency department, and patients are transferred for computed tomography (CT) scanning on a trauma transfer board. To reduce the possibility of secondary deterioration in spinal injury, it is desirable to maintain proper spinal immobilisation applied before hospital arrival through CT scanning. We propose to deactivate (inflate) the vacuum mattress upon arrival in the trauma bay, perform the needed survey and treatment, and reactivate (deflate) the vacuum mattress prior to transfer for CT scanning.

However, personal reports have indicated that a vacuum mattress may considerably reduce diagnostic quality. Recent studies indicate that immobilising head blocks both increase radiation and produce noise and artefacts that to some extent impair clinical judgement [8, 9]. Euler et al. recently demonstrated that whole body CT could be performed on trauma patients who are immobilised in vacuum mattresses without a relevant effect on the radiation dose or image quality, provided that the metallic valve in the vacuum mattress is not placed centrally, which is often the case [10]. In most of these cases, the device typically must be removed. Our theory was that modern immobilisations devices without metal parts may cause less artefacts and noise at acceptable radiation doses, and thereby facilitate the use of these devices through a CT scanning. The aim of our study was to investigate the effect of vacuum mattresses with plastic valves on CT artefacts, the radiation dose, and noise compared to a trauma transfer board and the spine boards currently used in our trauma system. As some immobilisation devices has large metal components that might generate artefacts on CT images, we also wanted to investigate the possibility of minimizing these during CT image reconstruction using metal artefact reduction algorithm currently available to us.

## Methods

To cover all technical and practical issues, the study group included members covering the treatment chain from the trauma site (paramedic), emergency department (anaesthesiologist and Head of Trauma), trauma CT-acquisition (radiographer), interpretation (radiologists), and radiation dose/quality control (physicist).

### Phantom and immobilisation devices

**Fig. 2:**
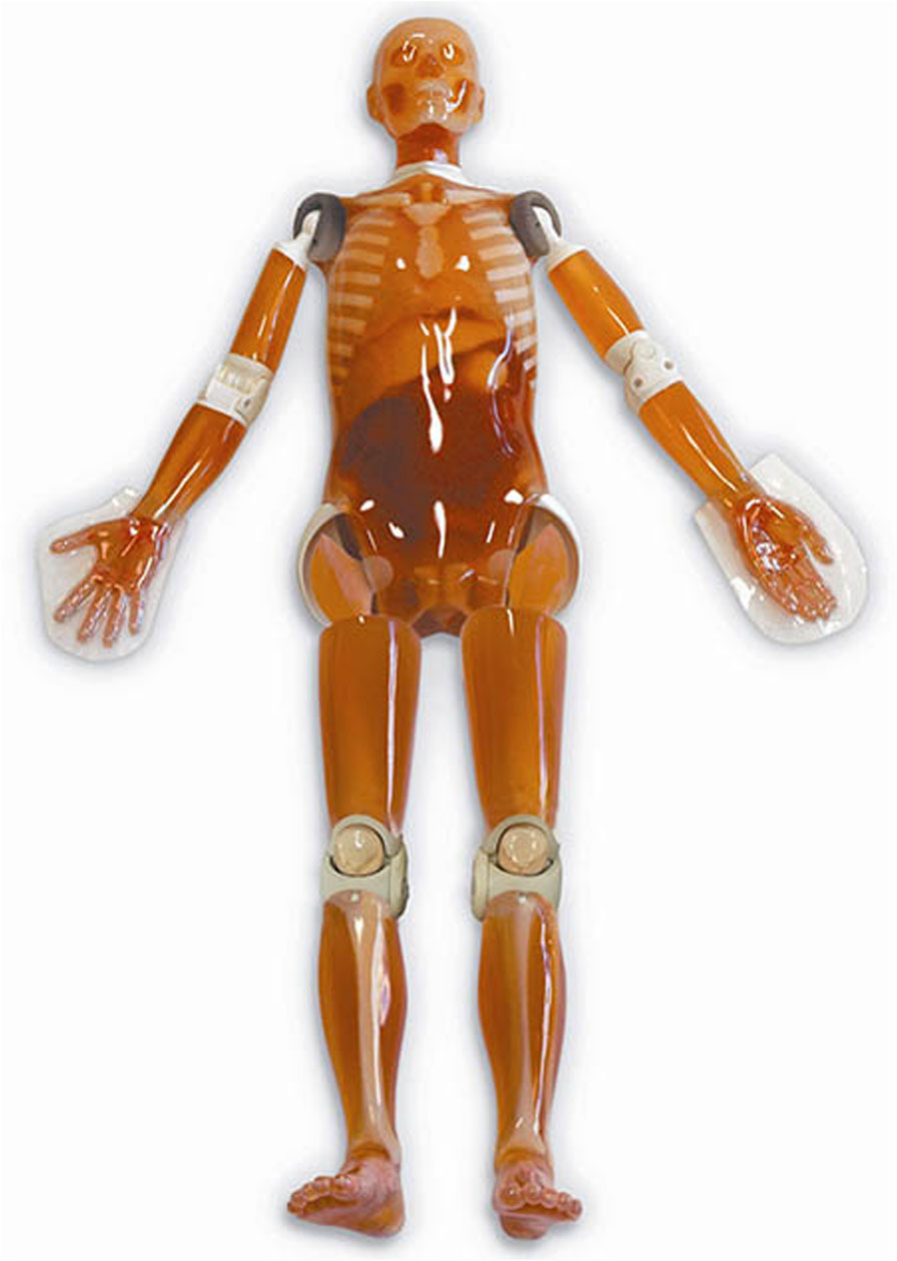
Anthropomorphic phantom PBU–60. **©** Kyoto Kagaku Co. LTD, Kyoto, Japan

To avoid irradiation of live patients, we used an anthropomorphic whole body phantom (PBU-60, Kyoto Kagaku Co. LTD, Kyoto, Japan). With a weight of 50 kg and height of 165 cm, the phantom resembles a small European adult patient with a synthetic skeleton, soft tissue, liver, kidneys, and vessels (Fig. 2). We performed scans using three different vacuum mattresses, two spine boards, and one trauma transfer board (Table 1). For these scans we also applied a standard semi-rigid cervical collar (Ambu Perfit Ace; Ambu, Ballerup, Denmark). In addition the phantom was scanned without any immobilisation device as a reference examination. All vacuum mattresses had a plastic valve placed in the chest/abdomen area. One spine board was made entirely of polyethylene plastic (Ambu Najo, Ferno, Brendale, Australia), while the other had metal connections (CombiCarrier II, Hartwell Medical, Carlsbad, California, USA). All vacuum mattresses were moulded around the phantom and activated (deflated) prior to CT scanning.

**Table 1:** List of immobilisation devices investigated

Case	Device	Name and vendor
1	No device	No device
2	Vacuum mattress 1	RedVac VM0991X01, Kohlbrat & Bunz, Radstadt, Austria
3	Vacuum mattress 2	Germa EasyFix, Ferno International, Wilmington, Ohio, USA
4	Vacuum mattress 3	Germa All in One Continental 90 cm, Ferno International, Wilmington, Ohio, USA
5	Spine board 1	CombiCarrier II, Harwell Medical, Carlsbad, California, USA
6	Spine board 2	Ambu Najo, Ferno, Brendale, Australia
7	Trauma transfer board	TraumaTransfer, Eson Comfort, Landeryd, Sweden
8	Spine board 1 with IMAR	CombiCarrier II, Harwell Medical, Carlsbad, California, USA

### CT protocol

We performed the scans on a 128-slice CT scanner (SOMATOM Definition Flash, Siemens Healthcare, Forchheim, Germany) with automatic exposure control (CAREDOSE 4D, Siemens) and automatic tube potential selection (CAREkV, Siemens). For head, neck, chest, and abdomen protocols, the reference tube current values were 390, 180, 75, and 206 mAs, respectively. For this phantom, the automatic programme chose 100 kV for the abdominal series and 120 kV for all other series. We positioned the arms along the trunk during the head and neck series and above the head during the chest and abdomen series. A metal artefact reduction algorithm (IMAR, Siemens) was available on our machine. IMAR is shown to reduce large metal artefacts in reconstruction of CT images [11]. We limited testing of IMAR to Spine board 1 (case 8, Table 1), being the only immobilisation device with large metal components.

As we were not accustomed to working with CT scans of phantoms, a reference scan was acquired and made available during the evaluation for comparison. We applied both soft tissue and bone reconstruction filters as well as iterative reconstruction (SAFIR, Siemens) for all series.

### Assessments

Two specialists in radiology independently reviewed all CT scans regarding artefacts and were blinded to the type of device. The order of the CT scans was randomised before reviewing.

**Fig. 3:**
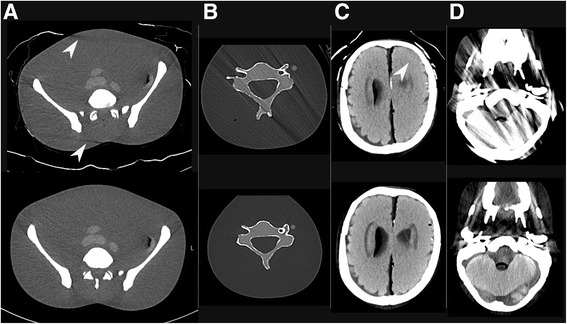
Examples of artefacts with reference images without artefacts below: **a** Artefact category 2. **b** Artefact category 3. **c** Artefact category 4. **d** Artefact category 1-3. White arrows indicate streak artefacts

The artefacts were categorised as no artefacts (category 1), artefacts not impeding clinical judgement (category 2), artefacts impeding clinical judgement (category 3), and artefacts rendering the investigation unsuitable for clinical judgement (category 4) (see examples of artefacts in Fig. 3a-d). Artefacts not visualised inside the phantom were classified as category 2. In the head series, however, even minor artefacts visualised inside the phantom were regarded as category 3, as they could obscure small but significant injuries. Agreement between the two radiologists was assessed using the kappa coefficient.

To assess the radiation dose, we used the Computed Tomography Dose Index Volume (CTDI_vol_) and dose length product (DLP). The effective dose for all CT series per device was estimated based on conversion factors from American Association Physicists in Medicine (AAPM) report 96 [12]. The effective dose for each scan was compared to the dose for the trauma transfer board which is the standard devise used in our trauma system.

To allow for noise evaluation, we performed each acquisition twice, as we exclusively used an anthropomorphic phantom. We estimated noise using the standard deviation of the difference in density (CT numbers) between the two acquisitions as follows:

$$ Noise=\frac{SD\left({I}_1-{I}_2\right)}{\sqrt{2}} $$

where I_1_ and I_2_ are the image intensities of series 1 and 2, respectively, and SD is the standard deviation.

## Results

The two radiologists agreed on 24 of 28 evaluations regarding artefacts (Table 2). The observed agreement was 0.86 with a kappa of 0.77. Fig. 3a-d shows examples of artefacts. Spine board 1 produced major artefacts due to its metal components. Vacuum mattress 3 also resulted in a category 3 artefact in the head series. Otherwise, the artefacts predominantly did not impair clinical judgement. We mainly observed subtle artefacts. The more prominent artefacts were primarily beam-hardening artefacts due to the folding of the mattress surface. The plastic valves in the vacuum mattresses did not create artefacts.

**Table 2:** Assessments of artefact category by two radiologists

Case	Device	Head	Neck	Chest	Abdomen
1	No device	1 / 1	1 / 1	1 / 1	1 / 1
2	Vacuum mattress 1	1 / 1	1 / 1	1 / 1	1 / 2
3	Vacuum mattress 2	3 / 1	2 / 2	2 / 2	2 / 2
4	Vacuum mattress 3	3 / 3	2 / 2	2 / 2	2 / 2
5	Spine board 1	4 / 4	3 / 3	1 / 1	1 / 1
6	Spine board 2	1 / 1	2 / 1	1 / 1	1 / 1
7	Trauma transfer board	1 / 1	1 / 1	1 / 1	1 / 2
8	Spine board 1 with IMAR	4 / 4	3 / 3	1 / 1	1 / 1

**Fig. 4:**
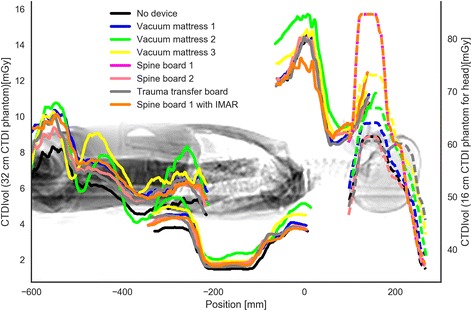
Each colour represents the CT radiation dose for the head, neck, chest, and abdominal series for each of the immobilisation devices investigated. The dotted lines represent the CT head series

There is a small increase in CT radiation dose output (CTDIvol) for scans acquired with an immobilization device compared to no device. Most notable increase in CTDIvol was in the head and chest series. For the head series, the dose output increased with 14 and 10% for spine board 1 and vacuum mattress 3 respectively. For the chest series, the two largest increases in dose output was 19 and 14% for vacuum mattress 2 and vacuum mattress 3 respectively (Table 3). The impact on the automatic exposure control from the devices is shown in Fig. 4.

**Table 3:** Radiation dose during the different series investigated

		CTDIvol [mGy]				DLP [mGycm]			
Case	Device	Head^a^	Neck	Chest	Abdomen	Head^a^	Neck	Chest	Abdomen
1	No device	50.2	11.2	2.7	5.7	929	253	96	240
2	Vacuum mattress 1	52.3	11.3	3.1	7.1	963	252	108	301
3	Vacuum mattress 2	54.3	12.4	3.7	6.7	983	299	135	297
4	Vacuum mattress 3	59.3	11.9	3.5	7.2	1094	260	123	293
5	Spine board 1	61.2	11.4	3.1	7.0	1111	252	113	291
6	Spine board 2	50.0	11.3	3.1	6.6	918	250	111	275
7	Trauma transfer	53.8	11.1	3.1	6.5	991	252	108	273
8	Spine board 1 with IMAR	61.2	11.4	3.1	7.0	1111	252	113	291

^**a**^ 16 cm CTDI phantom, all others 32 cm phantom

All scans with an immobilization device had an increased effective dose compared to trauma transfer board, except spine board 1 that was marginally lower (Table 4).

**Table 4:** Effective dose and mean image noise for all cases

Case	Device	Effective dose [mSv]	Effective dose compared to Trauma Transfer [%]	Mean image noise across all series [HU]	Mean image noise compared to Trauma transfer [%]
1	No device	8.4	−9	18.6	−2
2	Vacuum mattress 1	9.5	4	17.8	−6
3	Vacuum mattress 2	10.2	11	20.7	9
4	Vacuum mattress 3	10.0	8	19.5	3
5	Spine board 1	9.8	6	20.2	6
6	Spine board 2	9.1	−1	18.7	−1
7	Trauma transfer	9.2	0	19.0	0
8	Spine board 1 with IMAR	9.8	6	20.5	8

**Fig. 5:**
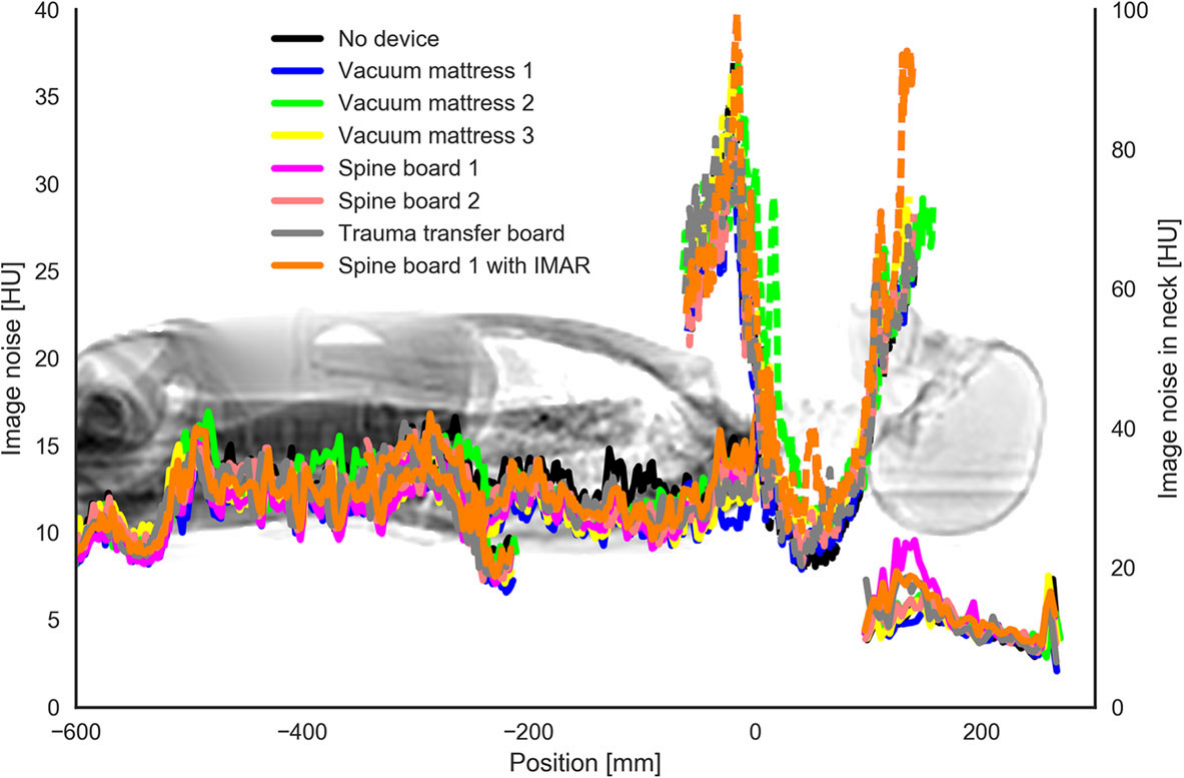
Each colour represents the image noise for the head, neck, chest, and abdominal series for each of the immobilisation devices investigated. The neck series exhibited more image noise (dotted lines) due to reconstruction using a bone filter

The noise profile across the CT series for each device (Fig. 5) shows only minor variations in image noise for each device. Mean image noise varied slightly. Compared to trauma transfer board the most notable difference was a 9 % increase in noise for vacuum mattress 2 and 6 % decrease for vacuum mattress 1 (Table 4).

The artefacts were categorised independently by the two radiologists as no artefacts (category 1), artefacts not impeding clinical judgement (category 2), artefacts impeding clinical judgement (category 3), and artefacts rendering the investigation unsuitable for clinical judgement (category 4).

## Discussion

We found substantial agreement between the two radiologists regarding artefact assessment. We did not observe any artefacts due to plastic valves. We observed beam hardening artefact (streak artefact) from the mattress canvas and combined beam hardening and metal artefacts from the metal components restricted to spine board 1.

Vacuum mattress 1 caused no artefacts that impeded clinical judgement.

Artefacts in head were subtle for vacuum mattress 2. Category 2 artefacts in the head series automatically became category 3 artefacts by definition. This can explain the difference in assessments between radiologist 1 and 2. Radiologist 1 specifically commented that the artefacts were minor. Apart from this finding, category 3 and 4 artefacts were limited to spine board 1 and vacuum mattress 3. Spine board 1 had metal components and produced significant beam-hardening artefacts, rendering the investigation unsuitable for clinical judgement. We were not able to correct this effect using metal artefact reduction.

Overall a slight increase in radiation dose was observed for almost all of the devices compared to the trauma transfer board. The largest increase in radiation dose output (CTDIvol) was 19% for vacuum mattress 2. The increase in dose output is most likely due to higher photon attenuation since the tested devices are larger and denser than the trauma transfer board, causing the automatic exposure control to increase the dose output. In our opinion, the increased radiation dose due to denser devices is small. The image noise profiles (Fig. 5) shows small variations across the devices. The variation in mean image noise is also small.

Overall, our results confirm those of Euler et al. [10] and indicate that plastic valves resolve the problem of artefacts produced by metallic valves.

We have not been able to identify any publications of neurological deterioration of spinal cord injuries, caused by emergency room handling. Indeed, a recent systematic review found no evidence for harm during the log roll manoeuvre, which may represent one of the situations with the greatest potential for spinal motion [13]. However, leading teaching systems like Advanced Trauma Life Support and Pre Hospital Trauma Life Support demand efforts to restrict spinal movement until a spinal injury has been ruled out [1, 2]. Therefore, such efforts will be made in most trauma systems aiming to provide maximum protection with minimum discomfort to the patient.

To prevent secondary deterioration in a possible spinal injury, it is desirable to maintain immobilisation throughout CT scanning. Furthermore, refraining from removing the immobilisation equipment may reduce the time consumption from arrival in the hospital to CT scans completed. Vacuum mattress 2 and 3 had to be deflated to free the arms (and then re-inflated in the new position) prior to chest/abdomen image acquisition. This adjustment, however, was not necessary with vacuum mattress 1. In addition to producing more artefacts than vacuum mattress 1, vacuum mattress 2 is broad and may not be able to pass into the CT gantry when applied to a large patient.

There are several limitations to our study. First, all scans were performed exclusively using one advanced CT scanner. Thus, we do not know the extent to which immobilisation devices would produce artefacts when older and less advanced machines are used. Second, our phantom represents a small adult patient. Automatic tube current modulation and automatic tube potential selection may make different choices with an average or large patient. The automatic tube potential selection was 100 kV for our phantom abdomen. For large patients, this value would normally be 120 kV or higher. Increasing tube potential is a technique to reduce artefacts from metal objects and will in similar fashion reduce streak artefacts from a dense mattress canvas. CT exposure is also increased for larger patients and may contribute to reduce streak artefacts. We therefore assume that an average or large patient would have less prominent CT artefacts. However, the reverse reasoning may imply more artefacts for smaller children. Third, we assessed only one scan per device. Different folding of the same device may theoretically induce differences in artefacts. Fourth: Although it was not possible to blind the interpreters completely to the generic type of device, they were blinded to manufacture type. Radiologists are not familiar to immobilisation devices in daily practise and we have assumed they therefore do not carry any bias on device type. Finally, our radiologists were not accustomed to evaluating CT scans of a phantom. Although it is realistic, the phantom still is not identical to a living patient, which may have affected the assessments.

Our results are based on a phantom study and may be viewed as an explorative feasibility study. A possible next step would be to conduct a prospective population-based study.

## Conclusions

Our results indicate that it is feasible to maintain some vacuum mattresses with plastic valves on trauma patients during CT scanning. The mattresses tested did not result in a considerably increased radiation dose or artefacts that impeded clinical judgement. One of the vacuum mattresses tested produced no artefacts that impeded clinical judgement.
